# Screening of Optimal Konjac Glucomannan–Protein Composite Gel Formulations to Mimic the Texture and Appearance of Tripe

**DOI:** 10.3390/gels10080528

**Published:** 2024-08-12

**Authors:** Qiang Zou, Yudie Liu, Linghui Luo, Yuyou Chen, Yuhan Zheng, Guilian Ran, Dayu Liu

**Affiliations:** 1 School of Food and Biological Engineering, Chengdu University, Chengdu 610106, China; zouqiang119@126.com (Q.Z.); liuyudie@stu.cdu.edu.cn (Y.L.); luolinghui@stu.cdu.edu.cn (L.L.); 17713417662@163.com (Y.C.); 15881060176@163.com (Y.Z.); 17366937100@163.com (G.R.); 2 Meat Processing Key Laboratory of Sichuan Province, School of Food and Biological Engineering, Chengdu University, Chengdu 610106, China

**Keywords:** protein isolate, KGM, composite gel, bionic tripe

## Abstract

This study aimed to develop a product that closely replicates the texture and appearance of tripe. The effect of three different proteins (soy protein isolate (SPI), pea protein isolate (PPI), and whey protein isolate (WPI)) at different protein levels and processing conditions (heating (90 °C, 1 h) followed by cooling (4 °C, 12 h) and heating (90 °C, 1h) followed by freezing (−18 °C, 12 h)) of konjac glucomannan (KGM) was analyzed. The optimal formulations for simulating tripe were screened by examining their similarity to real tripe in terms of texture, color, and sensory experience. The screened formulations were also subjected to a preliminary mechanistic investigation. The results show that all three proteins improved the gel’s textural properties to varying degrees. At the same concentration, the hardness and chewiness of the KGM/WPI composite gel were significantly higher than those of the other two KGM/protein composite gels, among which the composite gel obtained by adding 8% WPI and 5% KGM heating-frozen (FWK4) had the greatest hardness and chewiness of 4338.07 g and 2313.76, respectively, and the springiness differences in all of the composite gels were small. In addition, the addition of protein increased the whiteness of the hybrid gels, with WPI having the most significant effect on the whiteness of the composite gels (whiteness increased from 30.25 to 62.80 as the concentration of WPI increased from 0 to 10%). Freezing increased composite gel hardness and chewiness, but reduced gel springiness and whiteness. Cluster analysis showed that the composite gel obtained by heating–cooling 8% WPI and 5% KGM (WK4) was very similar to the real tripe in terms of chewiness and whiteness, and WK4 had the highest sensory scores for color, tissue morphology, tactile sensation, taste, and odor. The acceptability score in terms of tissue morphology reached 4.3. Meanwhile, the characterization results of WK4 indicate the presence of large junction areas in the gel network. Fourier transform infrared spectroscopy (FTIR) analysis, X-ray diffraction, and intermolecular force contributions indicated that the incorporation of WPI promoted integral interactions, and that hydrophobic interactions and disulfide bonding played a key role in the WK4 composite gel system. Moreover, scanning electron microscopy (SEM) also showed that the combination of WPI and konjac glucan resulted in a more compact gel structure. This study is informative for the development of the field of bionic tripe processing.

## 1. Introduction

Beef tripe is particularly popular in the Asian market due to its unique flavor and nutritional value [1]. With the rise of the plant-based market, non-meat protein products are also showing potential in the field of tripe [2,3]. However, relatively little research has been conducted on edible non-meat protein alternative to tripe [4], especially those products that accurately mimic the texture, color, and sensory experience of natural tripe. If a non-meat protein product could be developed to closely mimic the texture and appearance of real tripe, it would not only satisfy consumer demand for healthy and environmentally friendly plant-based food products, but also provide an edible experience similar to that of traditional tripe.

Gel simulation bionics is an advanced technology that uses polymer network systems to mimic the structure and function of biological tissues [5]. This technique has been widely used in the medical field for skin simulation for wound repair [6], cosmetic surgery [7], and in vitro release experiments [8]. For example, Wei et al. [9] developed a skin simulation material with moisturizing and anti-freezing properties using polyacrylic acid (PAA) and betaine; Zhang et al. [10] simulated the viscoelasticity of porcine skin using agarose gel; and Beatrice et al. [8] used a stabilized hydrogel to simulate the vascular flow pool of the cardiac diaphragm to study the release and distribution of drugs in vitro, and these studies have fully shown the wide practicality of gel simulation technology. If gel simulation bionic technology can be applied to the production of food products, it will help to develop new types of food to meet the diversified needs of the market and may have a positive impact on the food industry.

At present, most materials utilized in gel bionic technology include polyacrylamide [11], PAA [9], nanofibers [12], and other non-edible substances. This limitation hinders the application of gel bionic technology in the food industry. KGM is a natural polymer polysaccharide derived from konjac. KGM is safe for consumption, and it possesses remarkable thickening and gelling attributes; thus, KGM is extensively used in the fields of food and medicine [13,14]. However, current research is mostly focused on the KGM single system [14] or the complex polysaccharide system [15]. This system lacks the toughness and chewiness required to effectively simulate the oral chewing experience of meat. Moreover, this type of system is nutritionally simple. The ingredients are relatively simple. It has been found that protein/polysaccharide composite gel systems can make up for the limitations of single-component gels and enrich the nutrition of the gel through synergistic interactions between ingredients. Soy-protein isolate (SPI), pea-protein isolate (PPI), and whey-protein isolate (WPI) are commonly used proteins in biomimetic meat processing [16,17,18], and many studies have also combined them with KGM to simulate meat products. For example, Ran et al. [19] simulated the texture and structure of fish-ball by using a hybrid gel of KGM and SPI; Wang et al. [20] structured the gel network of PPI and KGM in order to mimic animal proteins; and Du et al. [21] applied a hybrid gel of WPI and KGM in 3D printing. But few studies have compared these three proteins’ properties to the macroscopic effect of KGM and apply them to the field of bionic tripe.

In addition, different processing conditions also have an effect on the properties of composite gels. It has been shown that the movement of water molecules in the hydrogel structure is restricted during freezing, which at the same time promotes polymer chain alignment or assembly, thus affecting the structure and texture of the gel [22,23,24]. Although this property is used in many applications such as the food, medicine, biology, and chemical industries [25,26], many of these studies were on single polysaccharide gels [27], konjac glucomannan/xanthan gum/sodium alginate composite gel [23], or soy protein-dextran conjugate gels [28]. There are few studies and applications on the effect of freezing on protein/KGM hybrid gels.

In this study, we compared the effects of three non-meat proteins (SPI, WPI, and PPI) on the texture and color of KGM composite gels at different protein levels and processing conditions. And using cluster analysis and sensory evaluation, these composite gel simulants were compared and screened with natural tripe stomach to obtain an edible composite gel formulation that simulated the texture, appearance, and taste of natural tripe. On this basis, the screened optimum gel formulation was subjected to rheological analysis, powder X-ray diffraction analysis, FTIR spectral analysis, contribution of intermolecular forces, and scanning electron microscopy analysis to preliminary explore its gel-forming mechanism in order to provide some help for the processing of non-meat protein products in the future.

## 2. Results and Discussion

### 2.1. Screening of the Most Suitable Recipe for Tripe Simulation

#### 2.1.1. Textural and Color Properties of Beef Reticulum

Cattle have four stomachs (including beef reticulum, rumen, omasum, and abomasum), all of which are edible and are particularly popular in East Asia, but they have very different textures. Among them, the reticulum is the popular product in China which is often referred to as “money tripe” by the Chinese, symbolizing wealth and prosperity, and its chewy and elastic nature makes it a favorite among Chinese consumers. Therefore, in order to cater for consumer preferences, the reticulated was selected as the simulation object in this study. In order to be able to more accurately simulate the textural appearance of tripe, this study determined the textural and chromatic characteristics of cooked fresh reticulum in an attempt to use this as a criterion for screening mixed gels prepared with different levels of concentration of the three proteins and processing methods. The characteristics of reticulum are shown in Table 1.

#### 2.1.2. Appearance Properties of Different Protein/KGM Composite Gels

**Figure 1:**
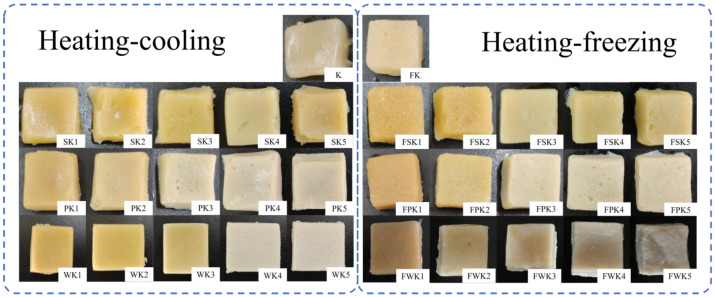
Appearance of different protein/KGM composite gels.

The effect of the different proteins on the overall appearance of the gels at different concentrations and processing conditions could be initially assessed by taking digital photographs of the gels (Figure 1). These photographs showed that all gels had an intact, solid appearance. Additionally, the samples in the K and FK groups appeared to be biased towards a transparent light yellow color, and the color of the mixed gels all changed from yellow to a color closer to the protein itself, with a gradual decrease in transparency as the amount of protein added increased from 2% to 10%. The surface of the frozen gels appeared to be wrinkled, and the lower the protein concentration, the more obvious the wrinkling phenomenon.

#### 2.1.3. TPA

Hardness, chewiness, and springiness are crucial factors in predicting the sensory texture of food. The data presented in Table 2 illustrate a pattern where the hardness and chewiness of the unfrozen composite gel initially increase and then decrease with the increase in protein concentration, indicating that lower levels of protein can interact with KGM molecules. This interaction leads to partial cross-linking and serves as a filler in the gel system, ultimately enhancing the textural properties of the gel. Conversely, an excessive concentration of protein can lead to water absorption and swelling, which impedes the formation of the KGM composite gel structure [29]. However, the change in springiness was not significant, which may be due to the fact that KGM is the main component that maintains the springiness of the gel in this concentration range and protein content is not a major factor affecting the springiness properties of the gel [21].

After the addition of protein, the network structure of the composite gel becomes more dense. Among the same concentration levels, the WK composite gel exhibits the highest hardness, followed by PK and SK. In particular, when the protein concentration is 2%, the hardness of the composite gel increases from 480.95 g to 552.27 g (SPI), 590.31 g (PPI), and 747.65 g (WPI). The WK composite gel has the highest hardness value, which is attributed to the low denaturation temperature of WPI. This phenomenon allows for more binding sites of the protein to interact with KGM [30], resulting in a tighter network structure. At a protein concentration of 8%, the hardness of the composite gel further increases to 696.23 g (PPI) and 1391.37 g (WPI). This trend can be explained by the reduced distance between protein molecules at higher concentrations, which leads to more collisions and stronger disulfide bonds, thereby densifying the network structure [31]. However, at this concentration, the hardness of the SK gel decreases to 465.62 g due to the high denaturation temperature of SPI, which prevents KGM from absorbing water and forming a network structure [32]. In addition, the large molecular weight of SPI compared with that of PPI and WPI increases the distance between KGM molecules and fills the gel pores, ultimately weakening the gel structure [33]. We also found that the composite gels composed of the three proteins did not show particularly significant differences in springiness, which may be consistent with the above results.

Comparing the composite gels before and after freezing (Table 3), the hardness and chewiness of frozen composite gels generally exceed those of unfrozen composite gels, aligning with Jiang’s research findings [23]. However, an interesting observation was made when analyzing the impact of protein concentration on frozen composite gels. As the protein concentration increased, the incremental increases in hardness and chewiness exhibited a downward trend. This trend was attributed to the hypothesis that higher protein concentrations might hinder KGM aggregation during freezing [28]. In particular, the hardness of frozen composite gels initially increased with higher protein concentrations and then decreased. For example, at a protein concentration of 2%, the hardness of frozen composite gels increased remarkably. At 8% protein concentration, the hardness of FWK and FPK composite gels increased, whereas that of FSK slightly decreased, which is consistent with the findings of unfrozen composite gels. Moreover, the chewiness of frozen composite gels followed a similar trend, that is, increasing and then decreasing with protein concentration. Notably, the chewiness of the FWK composite gels was consistently higher than that of the other groups. Springiness also varied with protein concentration, with FSK initially increasing and then decreasing, FPK being similar to PK, and FWK gradually decreasing as the protein concentration increased. Overall, the textural characteristics of the WK system are more akin to beef reticulum when compared with the other composite gels.

#### 2.1.4. Shear Force

**Figure 2:**
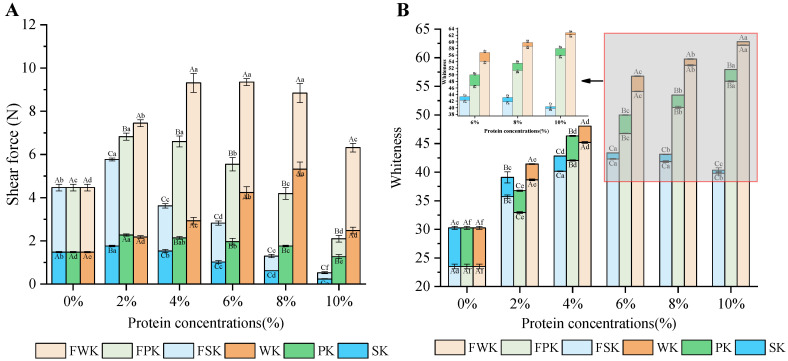
Effect of different protein concentrations on the shear force (**A**) and the whiteness (**B**) of the analog. Mean values with different letters are significantly different at *p* < 0.05. (SK: KGM is 5%, add 2–10% SPI, using heating–cooling method; FSK: KGM is 5%, add 2–10% SPI, using heating–freezing method; PK1: KGM is 5%, add 2–10% PPI, using heating–cooling method; FPK: KGM is 5%, add 2–10% PPI, using heating–freezing method; WK: KGM is 5%, add 2–10% WPI, process by heating–cooling; FWK: KGM is 5%, add 2–10% WPI, process by heating–freezing).

Shear force is an important indicator of the tenderness of gel food, and it can be used as a reference for subjective sensory evaluation during chewing. The shear force of unfrozen konjac composite gel initially increased and then decreased with increasing protein concentration (Figure 2A). Across all protein concentration levels, the shear forces of WK samples were consistently higher than those of the SK and PK samples. In particular, the shear force of the WK4 analog was 8.6 times greater than that of the SK4 analog and 3.0 times greater than that of the PK4 analog, respectively, indicating that WK performs better under high protein concentration conditions. This result can be attributed to the small molecular weight and low denaturation temperature of WPI, which not only fills the composite gel system, but also undergoes self-reactions and mutual reactions to enhance intermolecular forces, thereby improving gel properties [21]. Furthermore, the shear force of frozen composite gel was found to be higher than that of unfrozen composite gel. However, as the protein concentration in the frozen composite gel increased, the increment in shear force showed a downward trend, which is consistent with the texture analysis results. Notably, the incremental change in shear force was most pronounced in the SK composite gel. For example, at a protein concentration of 2%, the shear force of SK was 1.763 N, whereas that of FSK was 5.768 N, representing a 227.17% increase. At a protein concentration of 8%, the increase was 109.85%. This phenomenon may be attributed to the large molecular weight of SPI, which, as the protein concentration increases, not only fills the gel pores, but also increases the distance between KGM molecules and hinders their aggregation under freezing conditions [28].

#### 2.1.5. Color

The color of food is a crucial factor in sensory evaluation, and it strongly influences consumers’ purchasing decisions. Table 4 shows that the addition of protein notably affects the color of the analog, particularly the L* value. As the protein content increases, the brightness of SK initially increases and then decreases. In particular, when 10% SPI is added, brightness significantly decreases (*p* < 0.01). The high SPI concentration may lead to water absorption in the system, resulting in a dull color of the gel. The L* values of PK and WK increase with the increase in protein concentrations. With the addition of 10% WPI, the WK system exhibits a brightness value of 62.78, the highest among the three mixed gel systems, which indicates a better water-holding effect [34]. The a* value of all composite gel systems initially increases and then decreases. At a protein concentration of 2%, the a-value of all three composite gel systems reaches its maximum. Similarly, at a protein concentration of 6%, all composite gel systems had negative a-values, indicating a greener appearance, with PK exhibiting the smallest value, possibly because of the light green color of the PPI protein powder. The yellowness value (b* value) increases and then decreases as protein amount rises, peaking at 2% because of the reaction between KGM and protein carbonyl ammonia under hot alkali conditions, producing yellow substances [35]. Excessive protein concentration leads to the gel color leaning toward the protein’s natural hue. Among the various parameters observed, the b* value of WK exhibited the most significant decrease. With the addition of protein ranging from 2% to 10%, the b* value decreased from 23.32 to 0.74. The analysis shown in Table 5 reveals a significant decrease in the L* value of the frozen sample (*p* < 0.05) and a significant increase in the a* and b* values. Therefore, freezing deepens the color and reduces the brightness of the sample. In particular, when the protein concentration is low, the color change becomes more pronounced. This may be caused by the low protein concentration, which results in a poor water-holding capacity of the composite gel, making it migrate water from the center to the gel surface during freezing [36].

The results presented in Figure 2B demonstrate that the whiteness of the konjac composite gel is significantly influenced by the protein concentration. Compared with the single-KGM system, the whiteness of the konjac composite gel system shows a notable increase, with whiteness values rising alongside protein concentration. Notably, the whiteness value of 10% WDK is the highest among the tested concentrations. This phenomenon can be attributed to the inherently milky white color of WPI, resulting in a whiter appearance when combined with KGM to form a gel [37]. Overall, the addition of three types of plant proteins enhances the whiteness of the gel matrix. However, carefully selecting the optimal protein concentration when producing bionic beef reticulum is crucial to prevent color discrepancies that could impact consumer preferences.

#### 2.1.6. Correlation Analysis

**Figure 3:**
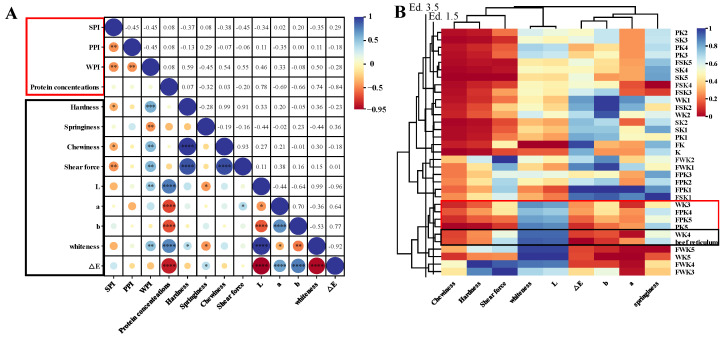
(**A**) Correlation analysis of textural properties (hardness, springiness, chewiness) and appearance characteristics (L*, a*, b*, whiteness, and ∆E) of different proteins (SPI, PPI, WPI) at different concentration levels. (“*” represents *p* < 0.05, “**” represents *p* < 0.01, “***” represents *p* < 0.001, “****” represents *p* < 0.0001; the “red box” represents the independent variable; the “black box” represents the dependent variable.); (**B**) Clustered heat map analysis of textural attributes (hardness, springiness, chewiness, shear strength) and appearance attributes (L*, a*, b*, whiteness, and ∆E) of mixed gels and tripe combined with different protein concentrations and processing methods (The “red boxes” represent the five groups of samples categorized with tripe at an Ed. of 3.5; the “black boxes” represent the groups of samples categorized with tripe at an Ed. of 1.5).

In order to determine the effect of different proteins at different concentrations on KGM, a correlation analysis was performed based on the results of hardness, springiness, chewiness, L*, a*, and b*, which are shown in Figure 3A. The correlation coefficients correspond to the different degrees and colors of circles in each rectangular box, where blue to red are positive to negative correlations. The correlation plots show that protein concentration correlates more strongly with texture and appearance metrics, while WPI correlates more strongly with texture and appearance metrics than SPI and PPI, which is consistent with the results above.

#### 2.1.7. Cluster Analysis

Cluster analysis is a method of grouping sample data based on similarity, which results in high similarity of samples within the same cluster and large differences between different clusters. In this study, we used systematic clustering to compare the similarities in texture and color between the gel simulants and the real reticulum in order to be able to screen the best protein–polysaccharide composite gel formulation to simulate reticulum. In a cluster analysis, Euclidean distance is a measure of similarity between samples; the smaller the distance, the higher the similarity. According to the experimental results, when the Euclidean distance was set to 3.5, the samples were divided into eight groups as shown in Figure 3B. Notably, WK3, WK4, PK4, PK5, FPK5, and beef reticulum were classified into the same group at this distance, suggesting that they have a high degree of similarity in texture and color.

While when the Euclidean distance was 1.5, only WK4 was clustered with the beef reticulum, which indicated that WK4 was more similar to the real tripe. Meanwhile, the results of the distribution of the color blocks showed that the samples in the WK4 group were highly consistent with the beef reticulum in terms of chewiness, hardness, whiteness, and brightness. This result compares with Du’s study that our prepared composite gel has improved in chewiness and hardness simulation [2].

To verify the accuracy of the cluster analysis and identify the closest analog to real reticulum, we plan further sensory evaluation studies to ensure the performance of selected formulations in practical applications.

#### 2.1.8. Sensory Evaluation

**Figure 4:**
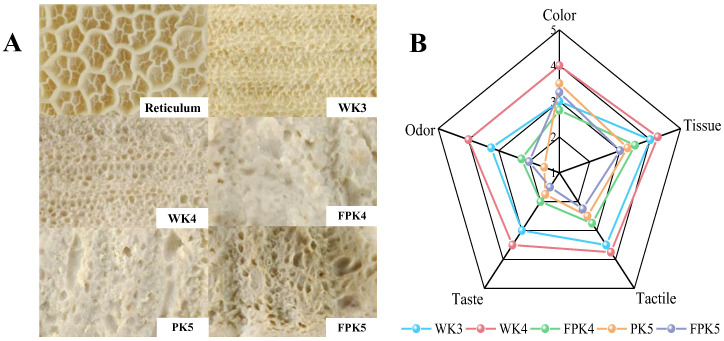
Appearance of composite gel simulant (**A**) with sensory evaluation radar chart (**B**).

Sensory evaluation can be an important basis for judging the quality of bionic meat to a certain extent. The WK3, WK4, FPK4, PK5, and FPK5 screened using cluster analysis were subjected to sensory evaluation in five aspects, namely, histomorphology, color, texture, tactile sensation, and odor, and sensory radar charts were produced (Figure 4B). The WK4 group had the highest overall acceptance level and was closest to the sensory profile of reticulum, while the FPK5 group had the lowest overall acceptance level. Meanwhile, the sensory scores of the WPI-added were overall higher than those of the PPI-added simulants, indicating that the WK hybrid gels were well accepted by the raters. Among them, WK4 had an acceptability score of 4.3 in terms of tissue, which proved that it was closest to the organization of reticulum; however, all five groups of samples had lower scores in terms of taste, with the highest-rated sample in the WK4 group scoring only 3.5, which proved that our screened mimics of tripe were still deficient in terms of taste.

It can also be seen from the digital photos of the reticulum and the five groups of samples (Figure 4A) that although the five groups of samples were all molded on the same reticulum mold, their mesh structures were quite different, with the WK group of samples having a relatively homogeneous mesh structure and the PK group of samples having a different size of mesh structure. The color of the simulants of the WK4 group was close to the color of reticulum, and there was rebound when pressed, and the entrance was chewy; while the simulants of the FPK5 group were close to white, but their color was grayish, and there was depression when pressed, and the entrance was crumbly and lack of chewiness, which was consistent with the results of the texture. The above results showed that the appearance and texture of the WK4 group were quite similar to that of the reticulum, and its odor and taste were within the acceptable range of the assessors, so the WK4 composite gel was judged to be the optimal simulation group by sensory tasting.

### 2.2. Characterization of Optimal Gel Formulations

#### 2.2.1. Rheological Analyses

**Figure 5:**
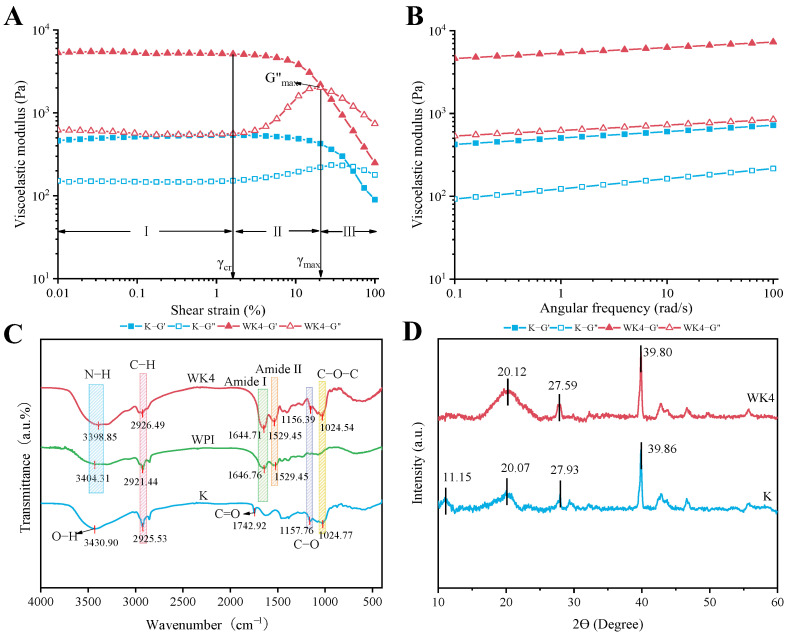
(**A**) Amplitude scans of group K and WK4. (“■” Group K energy storage modulus (G′); “□” Group K loss modulus (G″); “▲” Group WK4 energy storage modulus (G′); “△” Group WK4 loss modulus (G″)); (**B**) Frequency scans of group K and WK4 (“■” Group K energy storage modulus (G′); “□” Group K loss modulus (G″); “▲” Group WK4 energy storage modulus (G′); “△” Group WK4 loss modulus (G″)); (**C**) FTIR spectrograms of group WPI, K and WK4; and (**D**) XRD analysis of group K and WK4. (5% KGM gel in group K; whey isolate protein in group WPI; 8% WPI and 5% KGM composite gel in group WK4).

Figure 5A shows the results of the amplitude scanning of group K and group WK4, from which it can be seen that the curves of the two groups are in the range of γ_cr_ (strain 0–1%), and the energy storage modulus (G′) and loss modulus (G″) almost do not change with the amplitude strain, which is the linear viscous region (LVR) of the gels [38]. Meanwhile, within the linear viscoelastic region, G′ is larger than G″ in both K and WK4 groups, demonstrating that both the K and WK4 groups exhibit characteristics of solid gels [39], and both G′ and G″ of WK4 samples are significantly increased in comparison with those of the K group, which further confirms that the presence of WPI significantly improves the structural strength of composite WPI-KGM gels. In addition, G′ gradually decreased with further increases in strain (1%−100%), indicating the rupture of the inter-particle network structure. At the same time, G″ appeared to bulge at the strain amplitude γ_max_ with a maximum G″_max_ (Figure 5A). This phenomenon is due to the fact that excluded volume interactions can limit shear-induced particle motion [40].

The results of the frequency scans performed within the linear viscoelastic region of the gels are shown in Figure 5B, where the G′ of both groups of samples is much higher than G″, and both the G′ and G″ of the gels gradually increase with the increase in angular frequency. Meanwhile, the G′ and G″ of the WK4 group appeared to be significantly increased compared with that of the K group, indicating that the incorporation of the proteins led to the formation of a more compact network and structure within the system, which led to the storage of more capacity during the shear process [41].

#### 2.2.2. FTIR and XRD

The FTIR spectra of the gel samples of groups K and WK4 are shown in Figure 5C. Compared to group K and the WPI group, the WK4 group showed a red shift around 3400 cm^−1^ wave number, which is usually related to the formation of hydrogen bonding, which may be due to the participation of a N-H stretching vibration on the peptide bond of WPI in the formation of hydrogen bonding by KGM [42], resulting in a shift in the absorption peak to lower wave numbers. Meanwhile, the absorption peaks near 1646 cm^−1^ in the amide I region and 1529 cm^−1^ in the amide II region of the WK4 group, compared with those of the WPI group, did not undergo significant shifts, but their peak intensities were significantly increased, which further confirmed that the interactions within the protein molecule or between it and the gel network were strengthened [43]. And the peak intensity at 1024 cm^−1^ was significantly increased compared with that of the KGM group, which may also be due to the enhanced interaction between KGM and WPI. In addition, the acetyl carbonyl absorption peak (1742.92 cm^−1^) of KGM disappeared from the formed composite gel after the addition of WPI, suggesting that the acetyl group in KGM was inhibited in the presence of WPI, and the KGM molecule was transformed from a semiflexible linear chain to a convoluted structure, which resulted in better cross-linking with WPI to form a gel [44].

The XRD of Group K and WK4 are shown in Figure 5D, and the XRD profiles of the two groups of samples are relatively similar, with lower peak intensities and crystallinity, and both exhibit typical amorphous-dominated curves [45]. Compared with the samples in group K, the addition of WPI led to a change in the crystal structure of KGM, and the peak originally located at 2Ɵ = 11.15° disappeared, whereas the peak located at 2Ɵ = 27.93° was shifted to 27.59°, which could be attributed to the fact that the addition of WPI altered the interaction force between KGM molecules, leading to the expansion of the crystal lattice which in turn affected the position of the diffraction peaks [46]. The increase in the peak area around 2Ɵ = 20.07° and 2Ɵ = 39.86°, as determined using MIDI Jade 6.5 software, suggests that there is an increased interaction between WPI and KGM, which improves the ordering of the crystals [47].

#### 2.2.3. Analysis of the Degree of Contribution of Molecular Interactions

**Figure 6:**
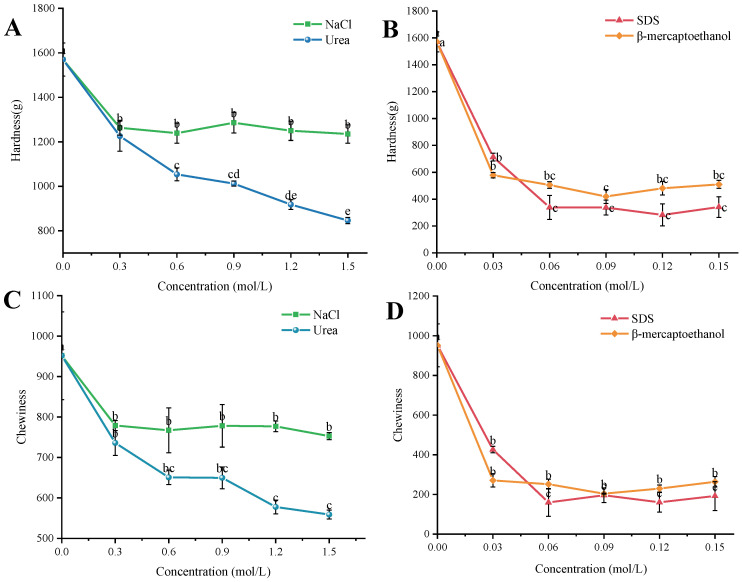
Analysis of the contribution of intermolecular forces in WK4 composite gels ((**A**,**B**) are the effects of different disruptors on the hardness gel; (**C**,**D**) are the effects of different disruptors on the chewiness of the gel) (a–e: Different lowercase letters indicate significant differences (*p* < 0.05)).

As shown in Figure 6, with the increase in the concentration of the ring-breaking agent, the hardness and chewiness of the group WK4 first significantly decreased and then plateaued, which is because the destructive agent destroys the intermolecular forces between the gels, and the hardness and chewiness will not be significantly changed when the corresponding forces are completely destroyed. Thus, the contributions of intermolecular forces can be determined by measuring changes in WK4 gel hardness and chewability, and provide a good basis for subsequent experiments. From Figure 6A,B, it can be seen that the hardness of the WK4 composite gel decreased slightly with the increase in NaCl concentration, which indicates that the electrostatic interactions are not the most dominant intermolecular force in the system; the hardness of the gel decreased continuously from the initial 1569.92 g with the increase in urea concentration, and the gel hardness of the gel was 846.03 g when the concentration of urea was 1.5 mol/L with the maximum loss rate was 46.11%. With the increase in SDS concentration, the gel hardness significantly decreased, reaching a maximum loss rate of 81.98%, indicating that hydrophobic interactions play an important role in the formation of the gel. With the increase in β-mercaptoethanol concentration, the hardness of the gel decreased significantly with the maximum loss rate of 73.27%, which indicates that disulfide bonding interactions also play an important role in the formation of the gel, but that the decrease is smaller compared to the hydrophobic interactions. As shown in Figure 6C,D, the chewiness of the gels also shows the same trend, which again proves that this method is practicable for the determination of inter-gel interactions.

The analysis of the factors affecting the texture of group WK4 showed that electrostatic interactions, hydrogen bonding interactions, and hydrophobic interactions have an important influence on the formation of WK4 composite gels, and the order of the strength of the forces in the gel is hydrophobic interactions > disulfide bonding interactions > hydrogen bonding interactions > electrostatic interactions. By analyzing the degree of contribution of various forces to the formation of composite gels, it can reflect the degree of influence of different forces on the network structure and other characteristics of the gel, which is of great theoretical significance for the construction and application of the gel system.

#### 2.2.4. SEM Observations

**Figure 7:**
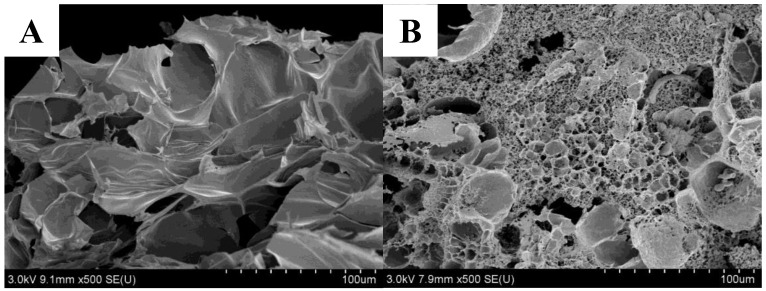
Microstructure of KGM gels (SEM images ×500) as affected by whey protein isolate (K, (**A**); WK4, (**B**)).

The microstructure of the gel after adding protein was observed and analyzed using scanning electron microscopy, compared to group K and the WPI group. The microstructure of group K displayed a discontinuous and irregular porous structure with larger pores and uneven distribution (Figure 7A), whereas group WK4 exhibited smaller pores, a continuous structure, and a denser composition (Figure 7B). This difference could be due to WPI’s role in water absorption and filling within the gel structure. Moreover, WPI undergoes denaturation under Ca(OH)_2_ and heating conditions, forming a three-dimensional network structure when combined with KGM [48]. The presence of protein also resulted in a rougher surface for the composite gel, which might be due to the attachment and reaction of WPI with KGM. These findings indicate that the internal interaction of the composite gel system is strengthened after protein addition, leading to improved stability, structure, and performance of the KGM gel.

## 3. Conclusions

In this study, we compared the effects of adding SPI, PPI, and WPI on the gel properties of KGM under cooling and freezing conditions. The addition of proteins enhanced the gel properties of KGM, improving hardness and chewiness, and the effect of WPI on the texture and chroma of the KGM composite gels was particularly significant; freezing facilitated the polymerization of the konjac/protein composite gels, which improved the gel properties, but also lowered the sensory scores. The best protein-polysaccharide gel formulation, WK4, which more accurately simulated tripe in terms of texture, appearance, and sensory experience, was screened by cluster analysis and sensory evaluation, and its characterization properties were investigated. The addition of WPI greatly affected the rheological properties, intermolecular forces, crystallinity, and microstructure of the KGM complexes. When the concentration of WPI was 8%, the energy storage modulus (G′) was enhanced compared to the single KGM gel, which was consistent with the results of the textural study, the strength of the hydrogen bonding and hydrophobic interactions was enhanced, and the crystallinity was increased, and the hydrophobic interactions and disulfide bonding interactions were the key to maintain the strength of the WK4 gel. The SEM images in the WK4 group showed that the gels formed a dense and continuous gel network. This study provides a theoretical basis for the development of biomimetic meat products using composite gels with different exogenous proteins and KGM.

## 4. Materials and Methods

### 4.1. Materials

Fresh beef reticulum (one of the tripe) was obtained from the meat market behind the North Gate of Chengdu University (Chengdu, China). KGM was provided by Hubei Johnson Konjac Technology Co., Ltd. (Ezhou, China); SPI was provided by Linyi Shan song Biological Products Co., Ltd. (Linyi, China); PPI was provided by Yantai Shuang ta Foods Co., Ltd. (Zhaoyuan, China); WPI was obtained from Volac Co., Ltd. (Felinfach, Lampeter, UK); Calcium hydroxide (Ca(OH)_2_) was obtained from Henan Wan bang Chemical Technology Co., Ltd. (Zhoukou, China).

### 4.2. Processing of Beef Reticulum

The fresh beef reticulum was washed with water to remove surface impurities (grass clippings, dust) and excess tissue (broken edges) with a knife and then was placed in a cooking pot and boiled in boiling water at 100 °C for 20 min. Take a part of it and cut it into regular pieces of 20 mm × 20 mm × 5 mm with a knife, and then cool it down to 25 °C and prepare it for use.

### 4.3. Preparation of Composite Gels

**Figure 8:**
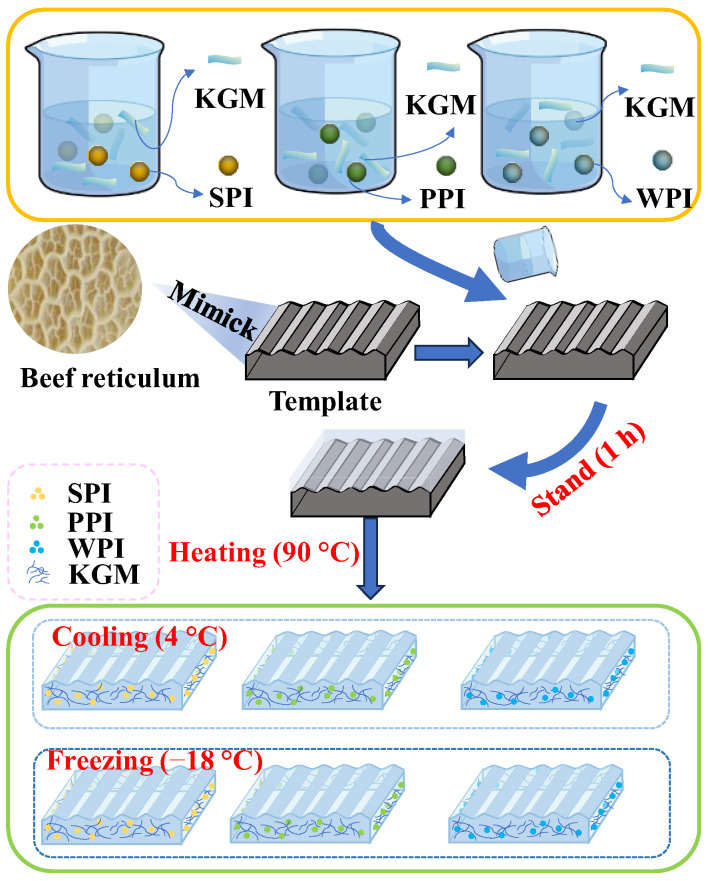
Flow chart of preparation of tripe analog.

The preparation of composite gels was performed according to the previous method, improving on it with pre-experiments [19]. PPI, WPI, and SPI were hydrated at different concentrations (0–10%, *w*/*w*) with 50% deionized water for 30 min. The proteins were dissolved completely. Then, the protein solution was added with 5% KGM and mixed evenly. (The reason why the protein concentration was fixed in the range of 0–10% and the KGM concentration was fixed at 5% was because after a large number of pre-experiments, it was proved that the protein/KGM mixtures in this range had a certain degree of fluidity, which could be conveniently molded. And they can both form stable solid gels after heating). Simultaneously, a certain amount of Ca(OH)_2_ (4 wt% of KGM mass) was added to make up for the mass difference with water (Figure 8 and Table 6). The mixture was placed into a silicone mold with a diameter of 20 mm × 20 mm × 5 mm, let stand for 1 h, and then placed in a water bath for heating and shaping (90 °C, 1 h). After the samples were recovered to 25 °C, the prepared samples were equally divided into two groups: one group was kept in the refrigerator at 4 °C for 12 h, which was the heating–cooling processing method, and the other group was kept in the refrigerator at −18 °C for 12 h, which was the heating–freezing processing method.

### 4.4. Texture Profile Analysis (TPA)

The prepared composite gel sample and beef reticulum were removed from the refrigerator and after recovery to 25 °C. The gel strength was measured using a TA-XT. plus texture analyzer (Stable Micro Systems Co., Ltd. (Godalming, Surrey, UK)). The testing conditions were as follows [2]: pre-test speed of 2 mm/s, test speed of 1 mm/s, post-test speed of 2 mm/s, cylindrical P/20 probe, trigger force of 15.0 g, and pressing depth accounting for 50% of the sample height. The results of the hardness, springiness, and chewiness were analyzed using the Exponent Connect software (Version 8, Stable Micro Systems Co., Ltd. (Godalming, Surrey, UK)), which is compatible with the texture meter. Each sample was measured 5 times in parallel. The reticulum was measured in the same way.

### 4.5. Shear Force

The shear force of the sample was measured using the A/MORS Meat of the texture analyzer (TA-XT. plus, Stable Micro Systems, UK). The experimental parameters were set as follows: A/MORS probe, pre-test speed of 1.0 mm/s, test speed of 1.0 mm/s, post-test speed of 10.0 mm/s, measured strain of 80%, trigger force of 15.0 g. The results of the shear force were analyzed using the Exponent Connect software (Stable Micro Systems, UK). Each sample was measured 5 times in parallel. The reticulum was measured in the same way.

### 4.6. Color

Referring to the method of Yao Li [37], a colorimeter (CS-220, Hangzhou Cai pu Technology Co., Ltd. (Hangzhou, China)) was used to measure the color of beef reticulum and composite gel simulants. The results are expressed in L* (lightness), a* (redness/greenness), b* (yellowness/blueness), whiteness value, and color difference value (△E). Each sample was measured 5 times in parallel. Reticulum was measured in the same way.

The formula for calculating the whiteness value is presented as follows:

(1) $$whiteness=100-\sqrt{{\left(100-L^{*}\right)}^{2}+a^{*2}+b^{*2}}$$

The calculation formula of color difference value (ΔE) is presented as follows:

(2) $$∆E=\sqrt{{\left(L^{*}-L_{0}^{*}\right)}^{2}+{\left(a^{*}-\alpha_{0}^{*}\right)}^{2}+{\left(b^{*}-b_{0}^{*}\right)}^{2}}$$

### 4.7. Sensory Evaluation

During a 5-day training session with 15 teachers and students (7 female and 8 male, mean age 29.5 ± 13.59 years) of the food program according to the International Standard method (ISO 8586-1 2012 [49]), the participants were familiarized with the odors of H_2_S and NH3 as well as the texture of tripe to understand the criteria for the assessment of odor and texture [50]. At the end of the training, a sensory evaluation panel evaluated the acceptability of samples screened using cluster analysis for color, tissue morphology, tactile sensation, taste sensation, and odor. At the completion of the training, each member received five de-identified samples placed in white plastic trays for sensory evaluation of color, histomorphology, taste, and odor, with effects between samples eliminated by referencing purified water. Acceptability of color, tissue morphology, tactile sensation, taste sensation, and odor was assessed using the 5-point descriptive scale, where score 1 corresponds to completely unacceptable and score 5 corresponds to very acceptable. The study was conducted at Chengdu University with the approval of the Ethics Committee, and all participants gave informed verbal consent for the analytical tests and product contents, including allergens. Participants had the right to withdraw at any time during the process.

### 4.8. Rheological Property

The textural properties of food products are usually associated with rheological properties [51]. The rheological properties of the composite gels were determined using an Anton Paar MCR302 rheometer using a PP 25 parallel plate fixture with a test gap of 5 mm. After loading the samples, the excess sample was removed and a layer of silicone oil was covered around the edges of the samples to prevent evaporation of water during the test. Strain scans were first carried out at 25 °C with a frequency of 1 Hz, an initial strain of 0.01%, a final strain of 100%, and a point count of 30 to determine the Linear Viscoelastic Range (LVR) and gel properties in this range. The LVR can help to pre-set consistent strain and stress values for further measurements. Subsequently, frequency scans were performed to characterize the viscoelastic properties. Frequency scanning: Frequency scanning (100–0.1 rad/s) was performed in the gel state at 25 °C, and a strain of 1% was chosen to ensure that it was within the linear viscoelastic region [52]. The samples were sealed with silicone oil to prevent evaporation.

### 4.9. Fourier Transform Infrared Spectroscopy (FTIR)

The molecular structure of the single KGM gel group K versus the screened optimum gel formulation group WK4 was analyzed using an infrared spectrometer (Spectrum 3, Agilent Technologies Inc., Santa Clara, CA, USA). About 2 mg of lyophilized sample was weighed and mixed thoroughly with 200 mg of potassium bromide powder, and the infrared spectrograms were obtained by scanning at 25 °C over a range of 4000–400 cm^−1^ with a resolution of 4 cm^−1^ and 32 scans [53].

### 4.10. X-ray Diffraction Analysis (XRD)

XRD is a technique to determine the crystalline structure of materials [47]. A powder X-ray diffractometer (D8, Bruker Co., Ltd., Karlsruhe, Germany) was used to test freeze-dried gels of a single KGM gel—group K—against a screened optimal gel formulation—group WK4. The test step size was 0.2°, the scanning rate was 2°/min, and the scanning angle range was 10°–60°.

### 4.11. Degree of Contribution of Intermolecular Forces

In order to investigate the contribution of intermolecular forces in the formulation of the best simulants, experiments were carried out with reference to Yu [54]. The gels were prepared using the preparation method of WK4 in Table 6, and the prepared WK4 was placed in different concentration gradients of denaturants at 4 °C for 12 h. The control group was soaked in distilled water. Changes in hardness and chewiness of the gel samples after soaking with different disruptors were measured using a TA-XT. plus texture analyzer (the operating parameters of the texture analyzer are the same as in 2.4) to investigate the extent to which electrostatic, hydrogen, hydrophobic, and disulfide bonds contribute to the intermolecular forces in the composite gel system. Each sample was measured five times in parallel. The concentrations of the disruptive agents are shown below:

(1) Electrostatic interactions: NaCl was used to break the electrostatic interactions in the gel system, and NaCl was added at concentrations of 0.3 mol/L, 0.6 mol/L, 0.9 mol/L, 1.2 mol/L, and 1.5 mol/L. The electrostatic interactions in the gel system were analyzed by using NaCl.
(2) Hydrogen-bonding interactions: Urea was used to destroy the hydrogen-bonding interactions in the gel system, and the urea was added at concentrations of 0.3 mol/L, 0.6 mol/L, 0.9 mol/L, 1.2 mol/L, and 1.5 mol/L.
(3) Hydrophobic interactions: Sodium dodecyl sulfate (SDS) was used to destroy the hydrophobic interactions in the gel system, and the concentrations of SDS were 0.03 mol/L, 0.06 mol/L, 0.09 mol/L, 0.12 mol/L, and 0.15 mol/L. The hydrophobic interactions in the gel system were also destroyed by the use of SDS.
(4) Disulfide bonding interactions: β-mercaptoethanol was used to destroy the disulfide bonding interactions in the gel system, and β-mercaptoethanol was added at concentrations of 0.03 mol/L, 0.06 mol/L, 0.09 mol/L, 0.12 mol/L, and 0.15 mol/L.

### 4.12. Scanning Electron Microscope (SEM) Observation

An S-4800 scanning electron microscope (HITACHI Co., Ltd., Tokyo, Japan) was utilized in this study. Freeze-dried samples of K and WK4 were fractured to reveal their cross-sectional areas and were subsequently coated with gold for 60 s prior to observation. The microstructure was then examined at an accelerating voltage of 3.0 kV and a magnification of 500 times.

### 4.13. Statistical Analysis

The results of the experiments are reported as the mean ± standard deviation. A one-way ANOVA was performed using SPSS 26 statistical software (IBM Corp., Armonk, NY, USA) to determine significant differences. A correlation analysis and cluster analysis of tripe and 32 gels prepared under different conditions were performed using Chiplot. Plots were plotted using Origin software (version 2018).

## Figures and Tables

**Table 1:** Texture and appearance characteristics of beef reticulum.

TPA	Hardness (g)	Springiness	Chewiness	Shear force (N)
	1344.37 ± 87.67	0.97 ± 0.01	1103.36 ± 49.92	7.31 ± 0.30
Color	L*	a*	b*	whiteness
	60.01 ± 0.61	−5.22 ± 0.97	2.96 ± 0.25	60.47 ± 0.50

**Table 2:** Effect of different protein additions on TPA of simulants under heating–cooling conditions.

Protein Type	Concentrations	Hardness (g)	Springiness	Chewiness
SPI	0%	480.95 ± 9.84 ^A,b^	0.87 ± 0.02 ^A,a^	227.92 ± 7.00 ^A,c^
	2%	552.27 ± 22.40 ^B,a^	0.89 ± 0.01 ^A,a^	299.71 ± 16.67 ^B,a^
	4%	500.03 ± 13.43 ^C,b^	0.87 ± 0.01 ^A,a^	272.43 ± 8.80 ^C,ab^
	6%	491.10 ± 8.04 ^C,b^	0.87 ± 0.01 ^A,a^	253.11 ± 10.41 ^C,bc^
	8%	465.62 ± 19.80 ^C,b^	0.89 ± 0.01 ^A,a^	256.66 ± 18.92 ^C,bc^
	10%	400.40 ± 29.41 ^C,c^	0.89 ± 0.01 ^A,a^	224.32 ± 14.62 ^C,c^
PPI	0%	480.95 ± 9.84 ^A,d^	0.87 ± 0.02 ^A,a^	227.92 ± 7.10 ^A,d^
	2%	590.31 ± 17.65 ^B,c^	0.85 ± 0.01 ^B,a^	295.77 ± 12.42 ^B,c^
	4%	614.89 ± 20.70 ^B,bc^	0.87 ± 0.01 ^A,a^	333.52 ± 12.48 ^B,b^
	6%	650.42 ± 24.34 ^B,ab^	0.87 ± 0.01 ^A,a^	346.78 ± 13.11 ^B,ab^
	8%	696.23 ± 31.94 ^B,a^	0.87 ± 0.02 ^AB,a^	372.48 ± 27.34 ^B,a^
	10%	490.35 ± 9.42 ^B,d^	0.88 ± 0.01 ^A,a^	266.15 ± 6.59 ^B,c^
WPI	0%	480.95 ± 9.84 ^A,d^	0.87 ± 0.02 ^A,a^	227.92 ± 7.10 ^A,e^
	2%	747.65 ± 59.51 ^A,c^	0.86 ± 0.02 ^B,a^	376.76 ± 27.48 ^A,d^
	4%	1021.97 ± 5.15 ^A,b^	0.86 ± 0.03 ^A,a^	526.17 ± 15.82 ^A,c^
	6%	1078.86 ± 5.85 ^A,b^	0.84 ± 0.03 ^A,ab^	533.09 ± 32.48 ^A,c^
	8%	1391.37 ± 2.65 ^A,a^	0.86 ± 0.01 ^B,ab^	739.63 ± 0.77 ^A,a^
	10%	1344.60 ± 15.42 ^A,a^	0.86 ± 0.02 ^B,c^	646.07 ± 6.83 ^A,b^

a–e: Different lowercase letters indicate significant differences (*p* < 0.05) between different additions of the same protein. A–C: Different capital letters indicate significant differences between different added amounts of the same protein (*p* < 0.05).

**Table 3:** Effect of different protein additions on TPA of simulants under heating–freezing conditions.

Protein Type	Concentrations	Hardness (g)	Springiness	Chewiness
SPI	0%	742.79 ± 22.98 ^A,cd^	0.89 ± 0.03 ^A,ab^	455.89 ± 31.88 ^A,b^
	2%	2055.32 ± 81.31 ^A,a^	0.91 ± 0.01 ^A,a^	1258.22 ± 12.01 ^A,a^
	4%	969.79 ± 26.38 ^C,b^	0.84 ± 0.02 ^A,bc^	474.29 ± 13.45 ^C,b^
	6%	810.71 ± 8.84 ^C,c^	0.80 ± 0.01 ^C,c^	360.13 ± 14.24 ^C,c^
	8%	688.07 ± 8.10 ^C,d^	0.79 ± 0.06 ^A,c^	325.00 ± 28.47 ^C,cd^
	10%	561.79 ± 6.53 ^C,e^	0.68 ± 0.01 ^C,d^	286.64 ± 10.68 ^C,d^
PPI	0%	742.79 ± 22.98 ^A,e^	0.89 ± 0.03 ^A,a^	455.89 ± 31.88 ^A,e^
	2%	1593.31 ± 73.63 ^B,c^	0.89 ± 0.02 ^A,a^	1025.44 ± 58.67 ^C,b^
	4%	2063.17 ± 98.95 ^B,a^	0.87 ± 0.01 ^A,a^	1204.70 ± 38.43 ^B,a^
	6%	1825.85 ± 45.54 ^B,b^	0.89 ± 0.02 ^A,a^	1147.82 ± 22.12 ^B,a^
	8%	1473.95 ± 21.95 ^B,c^	0.87 ± 0.04 ^A,a^	881.06 ± 77.45 ^B,c^
	10%	1062.31 ± 66.74 ^B,d^	0.86 ± 0.01 ^A,a^	577.79 ± 11.67 ^B,d^
WPI	0%	742.79 ± 22.98 ^A,e^	0.88 ± 0.03 ^A,a^	455.89 ± 31.88 ^A,f^
	2%	2058.88 ± 71.29 ^A,d^	0.85 ± 0.02 ^B,b^	1139.17 ± 42.79 ^B,e^
	4%	2863.26 ± 96.15 ^A,c^	0.86 ± 0.01 ^A,ab^	1669.18 ± 37.40 ^A,c^
	6%	3811.11 ± 181.49 ^A,b^	0.83 ± 0.02 ^B,b^	1987.82 ± 63.29 ^A,b^
	8%	4338.07 ± 46.15 ^A,a^	0.84 ± 0.01 ^A,b^	2313.76 ± 26.99 ^A,a^
	10%	2783.61 ± 127.93 ^A,c^	0.79 ± 0.01 ^B,c^	1444.36 ± 85.50 ^A,d^

a–f: Different lowercase letters indicate significant differences (*p* < 0.05) between different additions of the same protein. A–C: Different capital letters indicate significant differences between different added amounts of the same protein (*p* < 0.05).

**Table 4:** Effect of different protein additions on the color of simulants under heating–cooling conditions.

Index	Protein Type	0%	2%	4%	6%	8%	10%
L*	SPI	29.92 ± 0.24 ^A,e^	36.52 ± 1.05 ^A,c^	40.48 ± 0.02 ^C,d^	41.20 ± 0.06 ^C,a^	42.16 ± 0.15 ^C,a^	39.72 ± 0.43 ^C,b^
	PPI	29.92 ± 0.24 ^A,f^	34.13 ± 0.15 ^B,e^	44.05 ± 0.07 ^A,d^	48.45 ± 0.08 ^B,c^	52.05 ± 0.04 ^B,b^	57.37 ± 0.01 ^B,a^
	WPI	29.92 ± 0.24 ^A,f^	36.82 ± 0.04 ^A,e^	43.38 ± 0.10 ^B,d^	55.48 ± 0.29 ^A,c^	59.56 ± 0.07 ^A,b^	62.78 ± 0.02 ^A,a^
a*	SPI	−0.96 ± 0.45 ^A,c^	1.96 ± 0.26 ^B,a^	0.18 ± 0.01 ^B,b^	−0.93 ± 0.10 ^B,c^	−3.45 ± 0.01 ^B,d^	−5.20 ± 0.02 ^C,e^
	PPI	−0.96 ± 0.45 ^A,b^	2.06 ± 0.21 ^B,a^	−2.23 ± 0.07 ^C,c^	−3.89 ± 0.10 ^C,d^	−4.91 ± 0.06 ^C,e^	−4.52 ± 0.01 ^B,e^
	WPI	−0.96 ± 0.45 ^A,d^	4.14 ± 0.03 ^A,a^	2.14 ± 0.05 ^A,b^	−0.37 ± 0.15 ^A,c^	−0.73 ± 0.08 ^A,cd^	−0.88 ± 0.04 ^A,d^
b*	SPI	6.70 ± 0.35 ^A,d^	17.78 ± 0.59 ^B,a^	16.50 ± 0.23 ^B,b^	15.65 ± 0.18 ^A,b^	9.91 ± 0.58 ^A,c^	6.97 ± 0.28 ^A,d^
	PPI	6.70 ± 0.35 ^A,e^	18.33 ± 0.19 ^B,a^	15.54 ± 0.06 ^C,b^	11.89 ± 0.12 ^B,c^	10.03 ± 0.08 ^A,d^	5.38 ± 0.10 ^B,f^
	WPI	6.70 ± 0.35 ^A,c^	23.32 ± 0.06 ^A,a^	22.43 ± 0.20 ^A,a^	10.64 ± 0.96 ^C,b^	4.15 ± 0.12 ^B,d^	0.74 ± 0.26 ^C,e^

a–f: Different lowercase letters indicate significant differences (*p* < 0.05) between different additions of the same protein. A–C: Different capital letters indicate significant differences between different added amounts of the same protein (*p* < 0.05).

**Table 5:** Effect of different protein additions on the color of simulants under heating–freezing conditions.

Index	Protein Type	0%	2%	4%	6%	8%	10%
L*	SPI	23.09 ± 0.35 ^A,f^	31.82 ± 0.16 ^B,e^	36.38 ± 0.13 ^C,d^	39.82 ± 0.07 ^C,b^	40.50 ± 0.12 ^C,a^	38.83 ± 0.38 ^C,c^
	PPI	23.09 ± 0.35 ^A,f^	28.71 ± 0.14 ^C,e^	39.50 ± 0.59 ^B,d^	45.20 ± 0.05 ^B,c^	50.19 ± 0.23 ^B,b^	55.03 ± 0.15 ^B,a^
	WPI	23.09 ± 0.35 ^A,f^	34.35 ± 0.09 ^A,e^	41.50 ± 0.09 ^A,d^	52.23 ± 0.04 ^A,c^	58.53 ± 0.12 ^A,b^	62.09 ± 0.07 ^A,a^
a*	SPI	−0.63 ± 0.32 ^A,b^	4.29 ± 0.14 ^B,a^	3.78 ± 0.20 ^A,a^	−0.26 ± 0.09 ^A,b^	−3.07 ± 0.12 ^B,c^	−4.09 ± 0.43 ^C,d^
	PPI	−0.63 ± 0.32 ^A,b^	5.04 ± 0.20 ^A,a^	−1.97 ± 0.57 ^C,c^	−4.39 ± 0.06 ^C,d^	−3.84 ± 0.05 ^C,d^	−3.79 ± 0.15 ^B,d^
	WPI	−0.63 ± 0.32 ^A,c^	3.24 ± 0.27 ^C,a^	1.36 ± 0.10 ^B,b^	−0.62 ± 0.05 ^B,c^	−0.74 ± 0.02 ^A,c^	−0.83 ± 0.04 ^A,c^
b*	SPI	7.96 ± 0.63 ^A,f^	22.35 ± 0.43 ^C,b^	23.47 ± 0.18 ^A,a^	16.94 ± 0.37 ^A,c^	12.07 ± 0.08 ^A,d^	10.03 ± 0.76 ^A,e^
	PPI	7.96 ± 0.63 ^A,e^	23.65 ± 0.12 ^A,a^	17.16 ± 1.72 ^C,b^	12.23 ± 0.03 ^C,c^	10.34 ± 0.18 ^B,d^	7.86 ± 0.27 ^B,e^
	WPI	7.96 ± 0.63 ^A,d^	23.16 ± 0.21 ^B,a^	20.40 ± 0.17 ^B,b^	13.47 ± 0.10 ^B,c^	3.49 ± 0.18 ^C,e^	2.66 ± 0.04 ^C,f^

a–f: Different lowercase letters indicate significant differences (*p* < 0.05) between different additions of the same protein. A–C: Different capital letters indicate significant differences between different added amounts of the same protein (*p* < 0.05).

**Table 6:** Formulation of imitation tripe prepared with different non-meat proteins and konjac glucomannan.

Processing Conditions		KGM (%)	SPI (%)	PPI (%)	WPI (%)	Ca(OH)_2_ (wt%)	Water
Heating-Cooling	Heating-Freezing						
K	FK	5				4	94.8
SK1	FSK1	5	2			4	92.8
SK2	FSK2	5	4			4	90.8
SK3	FSK3	5	6			4	88.8
SK4	FSK4	5	8			4	86.8
SK5	FSK5	5	10			4	84.8
PK1	FPK1	5		2		4	92.8
PK2	FPK2	5		4		4	90.8
PK3	FPK3	5		6		4	88.8
PK4	FPK4	5		8		4	86.8
PK5	FPK5	5		10		4	84.8
WK1	FWK1	5			2	4	92.8
WK2	FWK2	5			4	4	90.8
WK3	FWK3	5			6	4	88.8
WK4	FWK4	5			8	4	86.8
WK5	FWK5	5			10	4	84.8

K: KGM is 5%, using heating–cooling method; FK: KGM is 5%, using heating–freezing method; SK1-SK5: KGM is 5%, add 2–10% SPI, using heating–cooling method; FSK1-FSK5: KGM is 5%, add 2–10% SPI, using heating–freezing method; PK1-PK5: KGM is 5%, add 2–10% PPI, using heating–cooling method; FPK1-FPK5: KGM is 5%, add 2–10% PPI, using heating–freezing method; WK1-WK5: KGM is 5%, add 2–10% WPI, process by heating–cooling; FWK1-FWK5: KGM is 5%, add 2–10% SPI, process by heating–freezing.

## Data Availability

The data presented in this study are available on request from the corresponding author.
